# Supplementary material for: Role of ACSBG1 in brain lipid metabolism and X-linked adrenoleukodystrophy pathogenesis: Insights from a knockout mouse model
Source: bioRxiv. 2024 Jun 20:2024.06.19.599741. Preprint. [Version 1] doi: 10.1101/2024.06.19.599741 (PMC11212999; doi:10.1101/2024.06.19.599741)
Supplement: Supplement 3 [file media-3.pptx]

## Slide 1
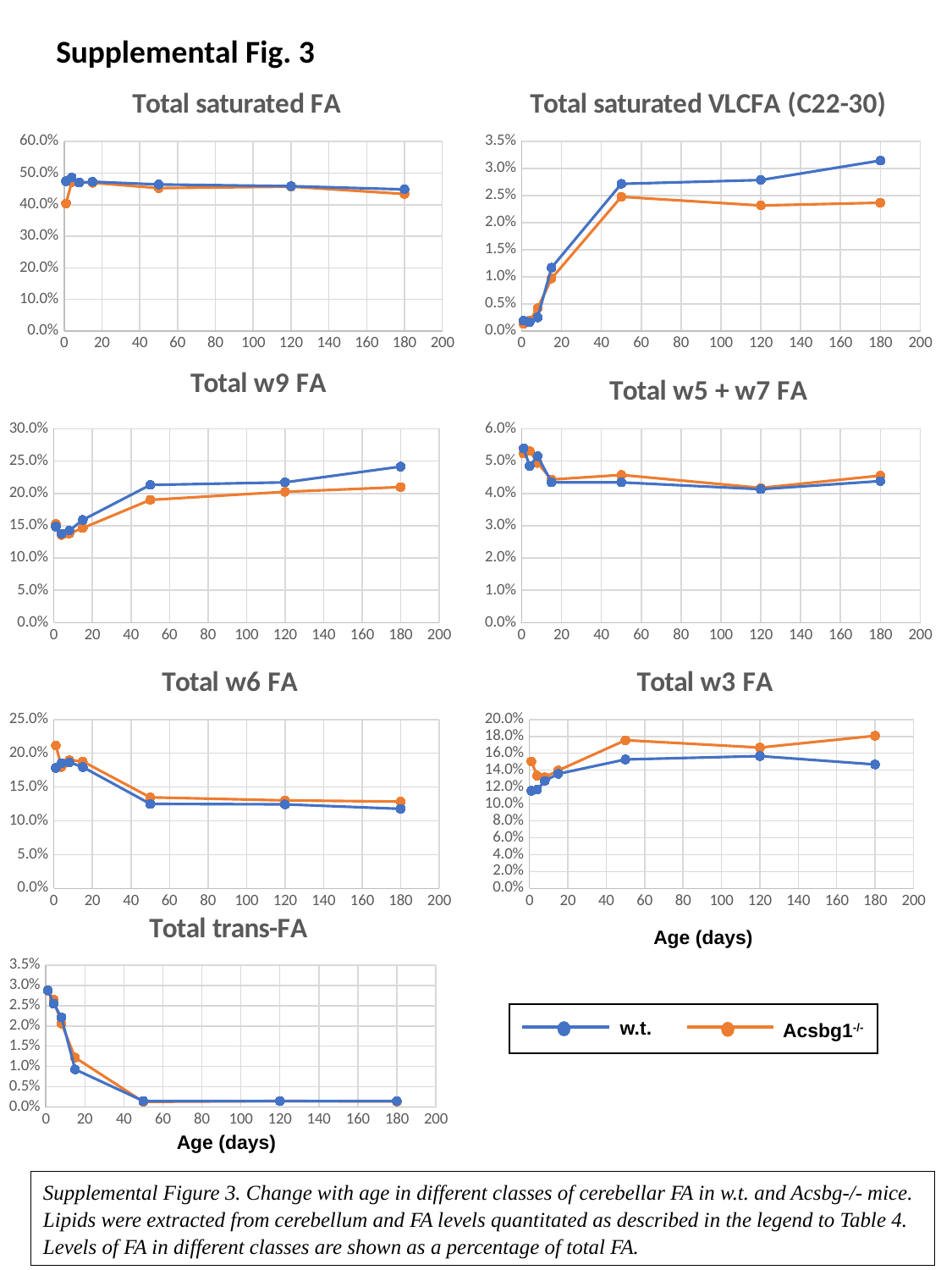

Supplemental Fig. 3
### Chart: Total saturated FA
| Category | w.t. | Acsbg1-/- |
|---|---|---|
### Chart: Total saturated VLCFA (C22-30)
| Category | w.t. | Acsbg1-/- |
|---|---|---|
### Chart: Total w9 FA
| Category | w.t. | Acsbg1-/- |
|---|---|---|
### Chart: Total w5 + w7 FA
| Category | w.t. | Acsbg1-/- |
|---|---|---|
### Chart: Total w3 FA
| Category | w.t. | Acsbg1-/- |
|---|---|---|
### Chart: Total w6 FA
| Category | w.t. | Acsbg1-/- |
|---|---|---|
### Chart: Total trans-FA
| Category | w.t. | Acsbg1-/- |
|---|---|---|Age (days)
w.t.
Acsbg1-/-
Age (days)
Supplemental Figure 3. Change with age in different classes of cerebellar FA in w.t. and Acsbg-/- mice. Lipids were extracted from cerebellum and FA levels quantitated as described in the legend to Table 4. Levels of FA in different classes are shown as a percentage of total FA.
